# Interaction effect of race-ethnicity and dementia on COVID-19 diagnosis among a national US older adult sample

**DOI:** 10.1192/bjo.2024.19

**Published:** 2024-03-14

**Authors:** Roger Wong, Jason Rafael Grullon

**Affiliations:** Department of Public Health and Preventive Medicine, Norton College of Medicine, SUNY Upstate Medical University, Syracuse, New York, USA; and Department of Geriatrics, Norton College of Medicine, SUNY Upstate Medical University, Syracuse, New York, USA; Norton College of Medicine, SUNY Upstate Medical University, Syracuse, New York, USA

**Keywords:** Cognitive dysfunction, coronavirus, dementia, ethnicity, racial groups

## Abstract

Older racial and ethnic minorities and older adults with dementia have an elevated COVID-19 risk, warranting research into the intersection between these two high-risk groups. We examined whether race-ethnicity moderates the association between dementia and COVID-19 diagnosis. Data were retrieved for 3189 respondents from a nationally representative prospective cohort sample of US older adults aged 65+ years. We analysed the effects of the interaction between race-ethnicity and dementia on COVID-19 diagnosis, after adjusting for sociodemographic factors, health and COVID-19 mitigation behaviours. The odds of COVID-19 diagnosis were significantly lower for Black older adults with dementia (adjusted odds ratio [aOR] = 0.07, 95% CI = 0.01–0.78, *P* = 0.03). In addition, dementia increased the odds of COVID-19 diagnosis among Hispanic older adults (aOR = 1.59, 95% CI = 0.12–21.29, *P* = 0.72), although this increase was not statistically significant. The interaction between race-ethnicity and dementia should be considered when assessing COVID-19 risk among older adults. Future research is needed to examine pathways through which dementia may interact with race and ethnicity to influence COVID-19 risk.

COVID-19 is an infectious disease that results from the SARS-CoV-2 virus.^1^ This is an important outcome of interest, given that it remains prevalent across the globe, and new strains continue to emerge. Relative to other age groups, older adults have the poorest prognosis and highest mortality from COVID-19. Provisional death counts from the Centers for Disease Control and Prevention showed that in 2022, approximately 94% of all US COVID-19 deaths were among individuals aged 50 years or older.^2^

A study using a national sample of US Medicare beneficiaries found that COVID-19 risk was significantly associated with race and ethnicity (White, Black and Hispanic).^3^ This finding may relate to Black and Hispanic older residents being more likely to reside in counties with high infection burden compared with White residents.^4^

Dementia is a syndrome with common symptoms that include loss of memory and thinking.^5^ Dementia is most commonly caused by Alzheimer's disease, for which a meta-analysis found higher risk among Hispanic (1.48 times), Black (1.34 times) and American Indian/Alaskan Native (1.14 times) populations compared with White older adults.^6^ Prior research using a national US older adult sample also found respondents with dementia to have significantly (129%) higher COVID-19 risk.^7^

Given the higher COVID-19 risk for older racial and ethnic minorities and older adults with dementia, the intersection between these two high-risk groups should be examined to determine whether public health intervention strategies should be modified. To our knowledge, our study is the first to use prospective national cohort data to assess whether the association between dementia and COVID-19 diagnosis varies by race and ethnicity among US older adults.

## Method

### Data source

We utilised the 2019 and 2020 waves of the National Health and Aging Trends Study (NHATS), a national (excluding Alaska and Hawaii) prospective cohort of 3189 US older adults aged 65+ years. NHATS data collection is ongoing, with data collected in-person annually. All COVID-19 variables were from the COVID-19 mailed supplement, and most responses were received in July 2020 (51%) or August 2020 (33%), prior to the emergence of any major variants of the SARS-CoV-2 virus.^8^ The authors assert that all procedures contributing to this work comply with the ethical standards of the relevant national and institutional committees on human experimentation and with the Helsinki Declaration of 1975, as revised in 2008. All procedures involving human subjects/patients were approved by the SUNY Upstate Institutional Review Board for the Protection of Human Subjects (1965041-1). NHATS interviewers obtained written informed consent from all respondents.

### COVID-19 diagnosis

COVID-19 diagnosis was developed from two self-reported questions: ‘Has a doctor or other health professional told you that you may have had COVID-19?’ (Yes, definitely; Yes, possibly; or No) and, ‘Have you had a positive test for COVID-19?’ (Yes or No). As in previous studies using this data on COVID-19,^2,4^ we defined a positive COVID-19 diagnosis as ‘Yes, definitely’ or ‘Yes, possibly’ from a doctor or a ‘Yes’ from a COVID-19 test.

### Dementia diagnosis

Dementia diagnosis was retrieved from 2019 using an NHATS algorithm with three cognition measures:^9^ (a) AD8 Dementia Screening Interview (cut-off point ≥2); (b) cognitive tests that evaluated memory (immediate and delayed 10-word recall), orientation (date, month, year and day of the week; President and Vice President naming) and executive functioning (clock drawing test) (cut-off point ≤1.5 s.d. below mean in ≥2 domains); and (c) self-report of an Alzheimer's disease or dementia diagnosis by a doctor. This binary variable was tested to have a good sensitivity of 65.7% and high specificity of 87.2%.^9^

### Race and ethnicity

Race and ethnicity were self-reported as either non-Hispanic White (hereafter, White), non-Hispanic Black (hereafter, Black), Hispanic or Other (Asian, American Indian, multiracial or unspecified).

### Covariates

Our regression model adjusted for sociodemographic, health and COVID-19 covariates. Sociodemographic variables included age, gender (male or female), highest level of education, total income, marital status, household size, metropolitan residence (metro or non-metro) and residential setting (community or residential care/nursing home).

Health variables included self-rated overall health (poor, fair, good, very good or excellent), body mass index, activities of daily living (ADL; no ADL limitations or at least one ADL limitation), proxy respondent, depression, anxiety, heart attack history, hypertension history, diabetes history and stroke history.

COVID-19 variables included three mitigation behaviours to prevent COVID-19 infection with binary responses (yes or no): handwashing (‘Frequently wash your hands or use sanitiser’), masking (‘Wear a face mask when going out’) and social distancing (‘Stay at least 6 feet away from people not living with you’).

### Analysis plan

We created an interaction effect between race-ethnicity and dementia to examine whether race-ethnicity moderated the dementia and COVID-19 relationship using a multiple logistic regression model. To minimise bias owing to missing data (12.5%), multiple imputation by chained equations was used to generate 100 imputed data files. Statistical analyses were performed in Stata 18,^10^ with two-tailed tests and α=0.05.

## Results

In our sample of 3189 older adults, most were White (76.1%), followed by Black (16.4%), Hispanic (4.1%) and other (3.4%). Mean age was 74.1 years, and a slight majority were female (57.8%). About 3.1% had received a positive COVID-19 diagnosis in 2020. There was high adherence to handwashing (97.2%), masking (96.7%) and social distancing (92.2%). Additional information about this COVID-19 supplement sample has been published elsewhere.^11^

**Fig. 1:**
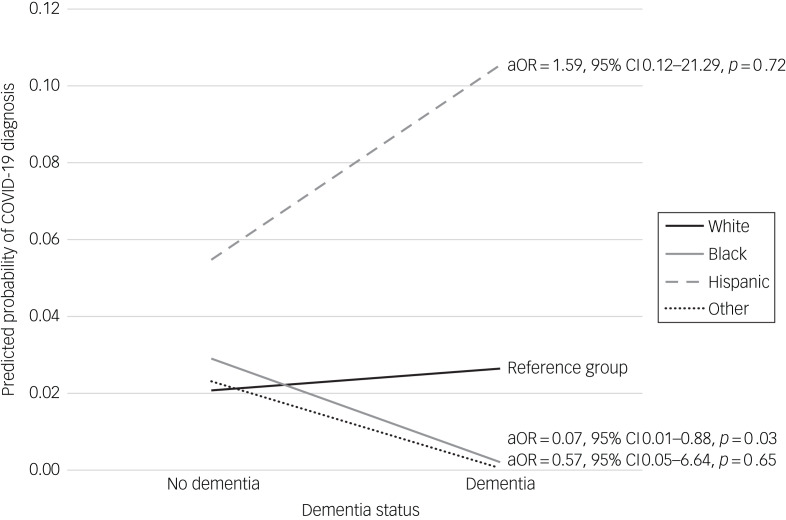
Effects of race-ethnicity and dementia interaction on adjusted predicted probability of COVID-19 diagnosis. Model is adjusted for dementia, race-ethnicity, age, gender, education, income, marital status, household size, metropolitan residence, residential setting, overall health, body mass index, activities of daily living, proxy respondent, depression, anxiety, heart attack history, hypertension history, diabetes history, stroke history, handwashing, masking and social distancing.

Our multiple logistic regression model found a statistically significant effect of the interaction between race-ethnicity and dementia on COVID-19 diagnosis [F(29,53) = 12.27, *P* < 0.001]; this analysis represented a US population size of 25 749 526 older adults after application of sampling weights. Compared with White older adults, the association between dementia and COVID-19 diagnosis was significantly different among Black older adults (adjusted odds ratio [aOR] = 0.07, 95% = CI 0.01–0.88, *P* = 0.03) but not Hispanic older adults (aOR = 1.59, 95% = CI 0.12–21.29, *P* = 0.72) or those in other groups (aOR = 0.57, 95% CI = 0.05–6.64, *P* = 0.65). Specifically, among Black older adults, those with dementia were significantly less likely to have COVID-19 compared with those without dementia (Fig. 1).

## Discussion

Our findings corroborate previous studies documenting that dementia may be associated with increased COVID-19 risk for certain groups.^7,12^ The directionality of such association, however, is further complicated when groups are stratified by race and ethnicity. Based on previous research,^7^ dementia increases COVID-19 risk for older adults through the following key mechanisms: having a low income, functional impairment in ADLs, and living in a residential setting/nursing home.

Black older adults with dementia were found in our analysis to have significantly decreased odds of COVID-19. By contrast, Hispanic older adults with dementia were found to have increased odds of COVID-19, although this difference was not statistically significant and may have been due to low statistical power. In addition, Hispanic older adults had higher odds of COVID-19, regardless of dementia status.

The pathways leading to our observed lower odds of COVID-19 among Black respondents with dementia are unclear, given the large variety in socioeconomic, health and community factors associated with COVID-19 diagnosis and adherence to COVID-19 mitigation behaviors.^3^ For example, although there is no existing research on this topic, it is possible that the relationship between dementia and employment status of older adults may vary by race and ethnicity. Our models were not able to adjust for employment; however, prior research using a large national US sample found that individuals who were employed had significantly (three times) higher odds of COVID-19.^13^

By contrast, Hispanic older adults with dementia may have had increased odds of COVID-19 because they had the lowest income and highest prevalence of ADL limitations compared with both older White and Black adults in our sample.^14^ In fact, a recent study indicated that relative to White older adults, although the odds of COVID-19 were higher for Asian (aOR = 1.68, *P* = 0.61) and Black (aOR = 1.13, *P* = 0.73) respondents, they were significantly higher for only Hispanic (aOR = 2.71, *P* < 0.01) respondents in the NHATS COVID-19 sample.^11^ Healthcare professionals should therefore take note that Hispanic older adults may have elevated odds of COVID-19, especially those with dementia, based on our current findings.

Allied health professionals should also aim to address barriers that may mitigate COVID-19 infection, such as the high cost of personal protective equipment and limited mobility.^7^ Future research is also needed to confirm our observed interactions between dementia and race-ethnicity with larger sample sizes, which will warrant collaborative approaches among older adults, caregivers, researchers and clinicians to ensure all racial-ethnic groups are adequately represented.^15^

Our study limitations are twofold. First, psychometric testing was not completed for any of our COVID-19 variables, owing to the novelty of the virus. Second, we constructed four broad categories of race and ethnicity owing to small sample sizes in subgroups. Despite these limitations, our study offers an important contribution by being the first to use prospective cohort data from a nationally representative US older adult sample to examine the interplay among race-ethnicity, dementia and COVID-19.

## Data Availability

This study used sensitive data; researchers may apply for access from NHATS (https://nhats.org/).
